# Mosaicism in Marfan Syndrome: Clinical Implications and Variant Allele Frequency Interpretation in a Narrative Review

**DOI:** 10.7759/cureus.106757

**Published:** 2026-04-09

**Authors:** Kota Arakawa, Hiroaki Ishida, Akiko Takashima, Takanobu Utsumi

**Affiliations:** 1 Department of Clinical Genetics Center, Toho University Medical Center Sakura Hospital, Sakura, JPN; 2 Department of Obstetrics and Gynecology, Toho University Medical Center Sakura Hospital, Sakura, JPN

**Keywords:** fbn1 gene, genetic counseling, marfan syndrome, mosaicism, variant allele frequency

## Abstract

Marfan syndrome (MFS) is an autosomal dominant disorder of the connective tissue caused by pathogenic variants in the FBN1 gene, characterized by cardiovascular, ocular, and skeletal involvement. Although some of these cases are considered sporadic, recent advances in next-generation sequencing (NGS) have revealed that certain cases might be attributable to parental mosaicism. Mosaicism, defined as the presence of genetically distinct cell populations arising from postzygotic mutations, retains important implications in diagnosis, phenotypic variability, recurrence risk, and genetic counseling. However, the interpretation of variant allele frequency (VAF) remains challenging and lacks standardization.

This narrative review summarizes our current knowledge on mosaicism in MFS, with a focus on VAF interpretation and its clinical implications. We performed a literature search using PubMed, focusing on studies published between 2010 and 2025, including relevant original and review articles addressing mosaicism in MFS.

Although VAF serves as a quantitative indicator of mosaicism, its interpretation is influenced by tissue distribution, mutation timing, and sequencing methodology. Peripheral blood VAF does not necessarily reflect variant burden in clinically relevant tissues, limiting its utility in predicting disease severity. Advances in high-sensitivity techniques, including NGS and digital droplet PCR, have improved the detection of low-level mosaicism, although multi-tissue analysis might be required for accurate diagnosis.

Mosaicism is an underrecognized but clinically important factor in MFS, particularly in apparently sporadic cases or when genotype-phenotype discordance is observed. Failure to detect mosaic variants may result in underestimation of disease severity, inaccurate risk assessment, and suboptimal genetic counseling. Clinicians should therefore consider mosaicism in atypical presentations, utilize high-sensitivity sequencing methods when appropriate, and assess parental status to refine recurrence risk. However, interpretation of VAF remains challenging due to tissue variability and methodological differences, and standardized frameworks linking VAF to clinical outcomes are lacking.

## Introduction and background

Marfan syndrome (MFS) is an autosomal dominant genetic disorder caused by pathogenic variants in the FBN1 gene located on chromosome 15q21.1 [1], and is characterized by multisystem involvement, primarily affecting the cardiovascular, ocular, and skeletal systems. MFS prevalence is estimated to be approximately 1 in 5,000 individuals, with no significant differences observed across sex or ethnic groups [1]. Among its clinical manifestations, cardiovascular complications (e.g., aortic aneurysm and aortic dissection) are the major determinants of prognosis, making early diagnosis and appropriate long-term management critically important [2]. Currently, MFS diagnosis is established based on the revised Ghent criteria, integrating clinical findings with genetic information, particularly FBN1 variants [3].

Approximately 25% of MFS cases are considered to arise from de novo pathogenic variants and are often recognized as sporadic cases without a family history [4]. Traditionally, such cases have been attributed to newly occurring mutations. However, recent advances in high-sensitivity genetic technologies, particularly next-generation sequencing (NGS), have revealed that a subset of these apparently sporadic cases might be explained by parental mosaicism [5]. While conventional Sanger sequencing might fail to detect low-level mosaic variants, the widespread adoption of NGS has allowed for the identification of variants with low variant allele frequencies (VAFs), thereby expanding the diagnostic spectrum of mosaicism.

The presence of somatic and germline mosaicism retains important implications for phenotypic variability, diagnostic accuracy, recurrence risk assessment, family screening, and genetic counseling strategies. In particular, low-level parental mosaicism might result in a higher recurrence risk than previously assumed, even in cases traditionally considered sporadic, necessitating the reconsideration of clinical management [6].

Although mosaicism in MFS is increasingly recognized, it warrants dedicated review because it directly impacts cardiovascular risk assessment, including life-threatening complications such as aortic aneurysm and dissection. Mosaic individuals may present with attenuated or atypical phenotypes, leading to underdiagnosis and inadequate surveillance. However, the frequency, clinical significance, and interpretation of VAF remain incompletely understood, and standardized frameworks for evaluation are lacking.

In this narrative review, we summarize the current understanding of somatic and germline mosaicism in MFS, with a particular focus on VAF interpretation, and discuss its diagnostic and clinical implications.

## Review

Methods

This narrative review was based on a targeted literature search using the PubMed database to identify relevant studies on MFS and mosaicism. The search period was defined from January 2010 to December 2025. The following keywords were used in various combinations: “Marfan syndrome,” “FBN1,” “mosaicism,” “somatic mosaicism,” “germline mosaicism,” and “gonosomal mosaicism.” Eligible studies included original articles and review articles published in English that addressed the genetic, diagnostic, or clinical aspects of mosaicism in MFS or related disorders. Studies were excluded if they were conference abstracts only, lacked sufficient relevance to the topic, or represented duplicate publications. The titles and abstracts of the retrieved articles were screened, followed by full-text assessment of potentially relevant studies. References of selected articles were also manually reviewed to identify additional relevant publications. Particular emphasis was placed on studies providing insights into VAF, detection methodologies, clinical implications, and genetic counseling.

Concept and mechanism of mosaicism

Mosaicism refers to the presence of two or more genetically distinct cell populations within an individual, resulting from postzygotic mutations [5,7]. It is broadly classified into somatic (variant confined to somatic cells), germline (variant present in gametes), and gonosomal (both somatic and germline involvement) mosaicisms [5,7]. Table 1 summarizes the classification and clinical features of mosaicism [7].

**Table 1:** Classification of mosaicism and its clinical implications. Based on Reference [7].

Classification	Definition	Clinical Features
Somatic mosaicism	Variant present in somatic cell lineages	Mild or atypical phenotype
Germline mosaicism	Variant present in gamete cell lineages	Recurrence in offspring from an unaffected parent
Gonosomal mosaicism	Variant present in both somatic and germline lineages	Affects both phenotype and recurrence risk

Mosaicism arises through mechanisms such as DNA replication errors, defects in DNA repair, chromosomal segregation errors, and genomic rearrangements. Mutations occurring early in embryogenesis tend to result in widespread mosaicism, whereas later mutations are often restricted to specific tissues (Table 2) [8,9].

**Table 2:** Mechanisms and developmental timing of mosaicism. Based on References [8,9].

Category	Description	Clinical Implication
DNA replication errors	Errors occurring during DNA replication	May lead to mosaic variants depending on timing
DNA repair defects	Failure of DNA damage repair mechanisms	Accumulation of postzygotic mutations
Chromosomal segregation errors	Abnormal distribution of chromosomes during cell division	Can result in mosaic aneuploidy or structural variants
Genomic rearrangements	Structural alterations such as deletions, duplications, or translocations	Contributes to genetic heterogeneity
Early embryonic mutations	Mutations arising in early developmental stages	Widespread distribution across multiple tissues
Late developmental mutations	Mutations arising in later stages	Restricted to specific tissues

Interpretation of variant allele frequency

VAF represents the proportion of sequencing reads containing a specific variant at a given genomic locus and is widely used as an indicator of mosaicism. In general, a VAF of approximately 50% is consistent with a heterozygous non-mosaic state, whereas values in the range of approximately 5% to 20% suggest mosaicism, and values below 5% indicate low-level mosaicism [10-12].

However, VAF is not an absolute measure and must be interpreted in context. It is influenced by multiple factors, including the type of tissue analyzed, the timing of the mutational event during development, and the sensitivity and depth of the sequencing method used. For example, peripheral blood VAF does not necessarily reflect variant burden in clinically relevant tissues such as the aortic wall or germ cells [13].

Furthermore, VAF alone cannot reliably predict disease severity or prognosis [13]. Individuals with low VAF may still develop significant cardiovascular complications, while those with higher values may exhibit milder phenotypes depending on tissue distribution [7,14,15]. Therefore, VAF should be interpreted as part of a broader clinical and genetic assessment rather than as a standalone indicator.

Diagnostic approaches

Sanger sequencing generally displays limited sensitivity for detecting low-level mosaicism, with difficulty identifying variants when the VAF is below approximately 15-20%. Therefore, certain cases of somatic mosaicism might yield false-negative results. In MFS, low-level mosaicism has been suggested in patients with mild or atypical phenotypes, and reliance on Sanger sequencing alone might lead to missed diagnoses [16].

Recently, the introduction of NGS has allowed for detecting VAFs of a few percent, improving the mosaicism identification rate in MFS. Furthermore, the use of ultra-deep NGS and digital droplet PCR (ddPCR) allows for the detection of extremely low-level mosaicism below 1%, being important for identifying parental mosaicism and assessing recurrence risk (Table 3) [17]. These techniques are applied when low-level mosaicism is suspected, such as in apparently unaffected parents of affected individuals, or in cases with atypical or mild phenotypes.

**Table 3:** Detection sensitivity of mosaicism in Marfan syndrome. Based on Reference [17]. ddPCR, digital droplet PCR; NGS, next-generation sequencing; VAF, variant allele frequency

Method	Detection Sensitivity (Approximate VAF)	Assessment
Sanger sequencing	~15-20% or higher	Low sensitivity
NGS (standard depth)	~1-5%	Moderate to high sensitivity
Deep NGS	~0.1-1%	High sensitivity
ddPCR	~0.01-0.1%	Ultra-high sensitivity

However, the sensitivity of these methods depends on the analyzed tissue. Even when variants are not detected in peripheral blood, they might be present in other tissues (e.g., the buccal mucosa, skin, or sperm). Therefore, when mosaicism is clinically suspected, the analysis of multiple tissues should be considered.

In addition, the FBN1 gene exhibits a broad mutation spectrum, including copy number variations (CNVs) and large deletions. Therefore, complementary methods, such as multiplex ligation-dependent probe amplification (MLPA), are desirable [18]. Accordingly, the diagnosis of mosaicism in MFS requires a comprehensive approach that takes into account both detection sensitivity and the choice of tissues for analysis.

Clinical significance of mosaicism in MFS

Phenotypic Variability

Mosaicism might result in mild or atypical phenotypes. Parents with subtle findings should not be considered unaffected without thorough evaluation [6].

Recurrence Risk

Parental mosaicism increases the risk of recurrence beyond that expected for de novo mutations, retaining significant implications for genetic counseling [6].

Family Screening

Even when parents appear unaffected, genetic testing should be considered in case a pathogenic variant is identified in the proband [6].

In this way, the importance of mosaicism in MFS extends beyond a rare genetic phenomenon and is directly linked to practical clinical concerns, including the re-evaluation of apparently sporadic cases, understanding of mild or atypical phenotypes, accurate recurrence risk assessment, and improvement in the quality of genetic counseling for families. Therefore, in MFS clinical management, particularly in cases that appear to be sporadic, actively considering the possibility of mosaicism is essential.

Clinical implications for management

Even in individuals with mosaicism in MFS, the risk of cardiovascular complications might persist; therefore, regular cardiovascular follow-up is essential. In particular, lesions directly affecting prognosis, such as aortic root dilation and aortic dissection, can occur even in cases with mild phenotypes, and underestimation of disease severity should be avoided [6].

In mosaic cases, the proportion and distribution of variant-bearing cells (tissue-specific mosaicism) influence organ-specific disease manifestations. Accordingly, assessing systemic risk based solely on VAF measured in peripheral blood is difficult. In addition, clinical manifestations may become more apparent with age, making long-term follow-up that accounts for temporal changes important [8]. These considerations are particularly relevant in the context of pregnancy, where hemodynamic stress may increase the risk of aortic complications. Even in individuals with apparently mild phenotypes or low peripheral blood VAF, careful cardiovascular evaluation and multidisciplinary management are essential during pregnancy and the peripartum period.

Therefore, even in cases where mosaicism is suspected, management should follow established guidelines, including regular echocardiographic assessment of aortic dimensions and, when appropriate, further evaluation using CT or MRI. Risk assessment should not rely solely on the apparent severity of the phenotype [19]. Furthermore, regardless of family history, family evaluation and genetic counseling based on the underlying genetic background are recommended.

From a clinical management perspective, surveillance strategies in patients with mosaicism should generally follow those established for classical MFS. In particular, aortic imaging intervals and modalities should not be relaxed solely based on an apparently mild phenotype, as serious complications such as aortic root dilation and dissection can still occur. Mild or atypical phenotypes in mosaic individuals should be interpreted with caution, as they may reflect limited tissue involvement rather than a truly lower risk profile. Therefore, clinical decision-making should incorporate both genetic findings and longitudinal imaging data. At present, there is no clear evidence to support the modification of surgical thresholds specifically for mosaic cases. Accordingly, established criteria for prophylactic aortic surgery in MFS should be applied, while individual factors such as family history, rate of aortic enlargement, and suspected tissue distribution of the variant should also be considered.

Future perspectives

Despite growing recognition of mosaicism in MFS, several challenges remain. Standardized frameworks for interpreting VAF have yet to be established. The relationship between VAF and clinical outcomes remains unclear, particularly in the context of tissue-specific mosaic distribution.

Advances in high-sensitivity sequencing technologies are expected to improve detection; however, distinguishing true low-level mosaicism from technical artifacts remains a challenge. Future research should focus on integrating genomic data with longitudinal clinical data to establish clinically meaningful thresholds.

In addition, improved strategies for multi-tissue sampling and minimally invasive testing may enhance diagnostic accuracy. Large-scale studies are needed to refine recurrence risk estimates and to develop evidence-based guidelines specific to mosaic cases.

## Conclusions

Mosaicism represents an important and increasingly recognized factor in the diagnosis and management of MFS. It contributes to phenotypic variability, influences recurrence risk, and presents challenges in genetic interpretation. Accurate assessment requires careful integration of VAF with clinical findings, tissue-specific considerations, and advanced genetic testing methodologies. Importantly, clinical decision-making should not rely solely on peripheral blood VAF but should incorporate longitudinal clinical evaluation and imaging findings.

Recognition of mosaicism is essential not only for improving diagnostic accuracy but also for guiding appropriate surveillance strategies, risk assessment, and genetic counseling in both affected individuals and their families.
